# Emotional eating in relation to psychological stress during COVID-19 pandemic: a cross-sectional study in faculty of medicine, Tanta University, Egypt

**DOI:** 10.1186/s12889-023-15177-x

**Published:** 2023-02-07

**Authors:** Walaa M. Shehata, Doaa E. Abdeldaim

**Affiliations:** Assistant professor of Public Health and Community Medicine, Faculty of Medicine, Tanta University, Tanta, Egypt

**Keywords:** Emotional eating, Psychological stress, COVID-19, Egypt

## Abstract

**Background:**

Stress, anxiety, and depression resulting from the COVID-19 pandemic as well as subsequent restrictive measures had a negative impact on eating behaviors. This study aimed to determine the emotional eating behaviors and the perceived stress level and to assess the relation between them among adults in the faculty of medicine during the late period of lockdown during the COVID-19 pandemic.

**Methods:**

This was a cross-sectional study among 580 persons from the faculty of medicine, at Tanta University, Egypt conducted over a period of one month during the last period of partial lockdown (October 2020). A self-administered structured questionnaire consisted of five parts used in the study (sociodemographic factors, COVID-19-related parameters, nutrition-related information during the pandemic, emotional eating scale, and perceived stress scale).

**Results:**

More than two-fifths of students, employees, and staff (45.2%, 45.5%, and 44.2%, respectively) stated that their weight increased as a result of the lockdown. 50.8% of students, 42.5% of employees, and 54.6% of staff were in the moderate emotional eating groups. Similarly, the majority of students, employees, and staff reported moderate levels of stress(84%, 80.8%, and 76.1% respectively). The total emotional eating score was positively correlated with the total perceived stress scores (r = 0.13, p = 0.001*).

**Conclusion:**

COVID-19 pandemic, particularly during the period of lockdown, had a negative impact on individuals’ psychological stress levels and levels of emotional eating behaviors.

## Introduction

Since 2020, Coronavirus infection (COVID-19) became a major public health problem that strongly disrupted people’s daily life all over the world, with tremendous effects on physical and mental health [1]. Trying to limit the spread of the virus disease, countries start to implement non-pharmaceutical interventions such as lockdowns and quarantine at the national and regional levels [2].

The social isolation and quarantine measures negatively affected the physical and mental state of individuals. Mental health consequences of the COVID-19 pandemic affected the whole population but more severely affected females, young adults, healthcare workers, and those of lower socioeconomic status as women are more likely to bear the extra burden of childcare as well as to work in sectors most affected by the pandemic [3, 4].

Emotional eating (EE) is defined as the tendency to overeat as a coping mechanism for regulating and reducing negative emotions, such as depression, anxiety, and stress [5]. EE is characterized by a failure to distinguish physiologic hunger sensations from the desire to use food as a coping strategy for negative emotion [6]. There is a relationship between people’s eating habits and their emotional status. Emotions like fear, anger, and sadness are very effective in changing the eating habits and behavior of individuals. Not only the biological need is required to eat, but also there is a psychological need. People eat more than usual if they feel anxious, overstressed, sad, angry or if they are under pressure [7]. As a result, eating becomes a method of distracting or escaping from negative affective experiences [8].

The stress, anxiety, and depression caused by COVID- 19 and its related consequences in the form of social isolation and absence of work and students life had a tremendous effect on people eating behaviors. At the start of the lockdown, panic affected people and make them excessively buy and store food and daily supplies. This panic is attributed to the uncertainty of the duration of the pandemic [9, 10].

This scenario with the changes that occurred in individuals eating habits, such as an increased number of cooked and consumed meals at home. Many individuals reported that they ate more than usual and had more unhealthy eating habits and behaviors. Most of them attributed these unhealthy dietary habits to their higher anxiety [11[13]. They prefer to consume energy-dense, tasty meals with mood-lifting effects which are often linked to high sugar content which in role leads to weight gain [14, 15].

Moreover, the COVID-19 pandemic plays a major role in triggering eating disorders behaviors and exacerbating their pre-existing symptoms. As it dramatically affects a person’s emotional behavior, causing severe stress and an increase in eating caused by the need to control one’s emotional state, which is reported in previous research [16–20]. Furthermore, restriction of individuals’ daily activities and their movements, lack of exercise practice, exposure to bad eating patterns present on social media in excessive ways, excessive emotional distress, and fear of infection [21].

This study aimed to determine the emotional eating behaviors and the perceived stress level and to assess the relation between them among adults in the faculty of medicine, at Tanta University, Egypt during the late period of lockdown during the COVID-19 pandemic.

## Subjects and methods

### Study design

this was an observational cross-sectional study.

## Target population and sampling

The study was conducted over a period of one month during the last period of partial lockdown in Egypt (October 2020). The target population was persons of the faculty of medicine, at Tanta University (250 students, 167 employees, and 163 medical staff). The number of students, employees, and staff was enumerated then the questionnaire was randomly distributed to the participants. Participants were selected through disproportionate probability stratified sampling technique. Study criteria included; (a) fourth-year students, employees, and staff in the Tanta faculty of medicine, (b) willing to participate (c) aged between 18 and < 60 years old. A total of 580 participants responded to our survey. A pilot study was carried out to determine the feasibility of the questionnaire before conducting the study.

## Sample size and sampling technique

Based on the worldwide prevalence of consuming unhealthy food during the COVID-19 lockdown (23.3%) and with a precision of 5% and 95% confidence level, power of study 0.8, a minimum sample size estimated was 270 subjects [10]. The total sample size was 580; 250 students, 167 employees, and 163 medical staff. Students in fourth grade were selected randomly. Sample size calculated by using EPi Info. objectives of the study were explained to the research participants. The response rate was 98%. This was a valid and reliable questionnaire (Cronbach’s Alpha 0.89).

## Study tool

A self-administered structured questionnaire consisted of five parts used in this study:

- The first part included items on sociodemographic factors (age, gender, marital status, education, residence, weight, BMI, employment, and family income). Based on self-reported weight and height, we calculated BMI (kg/m2).
- The second part was COVID-19-related parameters (change in family income, changing residency, infection by COVID-19, family member infected with COVID-19, type of quarantine).
- The third part was nutrition-related information during a pandemic. Questions related to the quarantine period included weight change because of lockdown (increase/decrease/no change), following a weight loss diet (yes/no), several meals and snacks per day, fast food intake (increase, decrease, no change), the frequency of eating or the urge to eat sweets (increase, decrease, no change), urge to drink coffee and tea and the amount of water intake.
- The fourth part was the emotional eating scale (EES): our study used a validated Arabic version of the Emotional Eating Scale (EES). It is a 25- item self-reported measure that assesses the desire to eat under the effect of negative emotions, such as resentful, Discouraged, Shaky, and Worn out,… [22]. The participants rate their answers to these items using a five-point Likert scale ranging from 0 (no desire to eat) to 4 (an overwhelming urge to eat). In our study, The EES had good test-retest reliability (r = 0.79, p < 0.001) and internal consistency Cronbach’s alpha was 0.81 [23]. The total score of the scale is calculated by summing the scores of all 25 items. The total score ranges from 0 to 100 and is divided into 0–25: a very healthy relationship with food, 25–46: moderate emotional eating, and 47–100: high emotional eating. The higher the score, the more emotional overeating among the participants.
- The fifth part was the perceived stress scale (PSS). It consists of ten items to quantify perceived stress [24]. This study used the validated Arabic version [25] to assess how participants perceived stress due to the pandemic and the quarantine. Participants rate their level of stress with a scale ranging from never (0) to very often (4). Each item was answered on a five-point Likert scale, with scores ranging from 0 (rarely) to 4 (almost always), with higher scores indicating more severe perceived stress [26]. The summed score is 40 and divided into 0–13 considered low stress, 14–26 considered moderate stress and 27–40 considered high perceived stress.

### Statistical analysis

Statistical Package for Social Sciences (SPSS) (version 16.0, IBM, Armonk, NY) was used to analyze the data of the study. For quantitative data mean ± SD was calculated. Descriptive presentations (n, %) were done for the qualitative data of the study. For comparison between chi-sq (x^2^), one-way ANOVA(F) was performed. The correlation was done between EES and PSS using the Pearson correlation test (R). To identify predictors of emotional eating, linear regression was used (adjusted and unadjusted). The level of significance was p < 0.05.

## Results

Table 1 shows the sociodemographic characteristics of the groups analyzed. The majority of students (97.2%) were between the ages of 18 and 25. 38.3% of employees were between the ages of 35 and 45, while nearly half (47.2%) were between the ages of 25 and 35. Females constituted 72.5% of students, 50.3% of employees, and 56.4% of staff. As regards educational level, most students (98.4%) were undergraduates. On the contrary, the majority of employees and staff (84.4% and 98.2% respectively) had a bachelor’s degree or higher. According to residency, 52.7% of employees were from rural areas, compared to 62.4% of students and 71.2% of staff who were from urban areas. In contrast to the 86.8% of married employees and 84% of married staff, 98.8% of students were single. More than half of students, employees, and staff had enough income (67.6%, 59.3%, and 60.7%, respectively). The mean of weights of staff, employees, and students were (75.4, 77.9, and 78 respectively). Consequently, The mean BMI for students, employees, and staff was 24.9, 27, and 26.8 kg/m^2^. In terms of COVID-19-related parameters, the majority of participants reported no change in family income or place of residence. There are more than half of students, employees, and staff who are not infected with COVID-19 (71.2%, 68.9%, and 74.8%, respectively), as well as their family members (50%, 65.3%, and 51.5% respectively). 28% of students reported infection in their father or mother, whereas 24% of employees and 32.5% of staff reported infection in their entire family. Self-quarantine was reported by 41.2% of students, 27.5% of employees, and 36.2% of staff. Table 2 shows the nutritional-related information among participants during a pandemic. More than two-fifths of students, employees, and staff (45.2%, 45.5%, and 44.2%, respectively) stated that their weight increased as a result of the lockdown. 72.8 of students, 79% of the employee, and 76.9%of staff did not follow a weight loss program. 42.4% of students consumed more meals and snacks daily, compared to 46% of employees and 46.1% of staff who consumed the same amount. Additionally, fast food consumption increased for 36.8% of students while being constant for 38.9% of employees and 36.8% of staff. In 46% of students, 42.5% of employees, and 39.3% of staff, the frequency of consuming or the desire to eat sweets increased. Coffee and tea consumption rose among 53.6% of students, 46.7% of employees, and 53.4% of staff. Moreover, the amount of water increased in 54.4% of students, 49.7%of employees, and 55.8% of staff. Table 3) represents the relationship between the emotional eating scale and perceived stress scale with employment. 50.8% of students, 42.5% of employees, and 54.6% of staff were in the moderate emotional eating groups. Similarly, the majority of students, employees, and staff reported a moderate level of stress(84%, 80.8%, and 76.1% respectively). The correlation between emotional eating score and perceived eating, sex, BMI, and weight were shown in Table 4. The total emotional eating score was positively correlated with the total perceived stress scores (r = 0.13, p = 0.001*) and weight(r = 0.11, p = 0.007*). Multiple linear regression analysis revealed that marital status (B = 0.3, p = 0.02*) without adjustment, and perceived stress scale score (B = 0.2, p = 0.006*) were positively correlated with emotional eating scores both adjusted and not adjusted as shown in Table 5.

**Table 1:** sociodemographic data and COVID-19-related parameters among the studied groups

Parameters	Employment			Test of sig	p
	Student N = 250	Employee N = 167	Staff N = 163		
**Age** :					
18-<25y	243(97.2%)	10(6%)	1(0.6%)		
25-<35y	6(2.4%)	57(34.1%)	77(47.2%)	X^2^ = 51.7	0.0001*
35-<45y	1(0.4%)	64(38.3%)	54(33.1%)		
≥ 45y	0(0%)	36(21.6%)	31(19%)		
**Sex** :					
Male	188(72.5%)	83(49.7%)	71(43.6%)	X^2^ = 49.1	0.0001*
Female	62(24.8%)	84(50.3%)	92(56.4%)		
**Educational level**:					
Undergraduate	246(98.4%)	26(15.6%)	3(1.8%)	X^2^ = 46.4	0.0001*
Graduate or higher	4(1.6%)	141(84.4%)	160(98.2%)		
**Residence**:					
Urban	156(62.4%)	79(47.3%)	116(71.2%)	X^2^ = 20.3	0.0001*
Rural	94(37.6%)	88(52.7%)	47(28.8%)		
**Marital status**:					
Married	3(1.2%)	145(86.8%)	137(84%)	X^2^ = 40.4	0.0001*
Single	247(98.8%)	22(13.2%)	26(16%)		
**Income**:					
Not enough	52(20.8%)	55(32.9%)	15(9.2%)		
Enough	169(67.6%)	99(59.3%)	99(60.7%)	X^2^ = 54.2	0.0001*
Enough and saving **Weight**	29(11.6%) 75.4 ± 13.7	13(7.8%) 77.9 ± 12	49(30.1%) 78 ± 12.7	F = 2.7	0.07
**BMI**	24.9 ± 3.7	27 ± 4.1	26.8 ± 3.4	F = 22.1	0.0001*
**COVID-19 parameters**					
**Change in Family income**:				X^2^ = 4.3	0.4
No	157(62.8%)	106(63.5%)	117(71.8%)		
Yes, decreased	76(30.4%)	52(31.1%)	38(23.3%)		
Yes, increased	17(6.8%)	9(5.4%)	8(4.9%)		
**Changing Residency**:					0.3
Yes	34(13.6%)	15(9%)	22(13.5%)	X^2 ^= 2.3	
No	216(86.4%)	152(91%)	141(86.5%)		
**Infected by COVID-19**:				X^2^ = 1.5	0.5
No	178(71.2%)	115(68.9%)	122(74.8%)		
Yes	72(28.8%)	52(31.1%)	41(25.2%)		
**Family member infected by COVID-19**:				X^2^ = 10.4	0.005*
No	125(50%)	109(65.3%)	84(51.5%)		
Yes	125(50%)	58(34.7%)	79(48.5%)		
**Specify member**:				X^2^ = 15.2	0.0001*
Father or Mother	70(28%)	18(10.8%)	26(16%)		
Extended family	55(22%)	40(24%)	53(32.5%)		
**Type of quarantine**:				X^2 ^= 12.3	0.02*
Medical isolation	22(8.8%)	12(7.2%)	20(12.3%)		
Self -quarantine	103(41.2%)	46(27.5%)	59(36.2%)		

**Table 2:** Nutrition-related information during COVID-19 lockdown

Parameters	Employment			X^2^	p
	Student	Employee	Staff		
**Weight change because of lockdown**:					
Increase	113(45.2%)	76(45.5%)	72(44.2%)		
Decrease	49(19.6%)	25(15%)	32(19.6%)	1.9	0.7
No change	88(35.2%)	66(39.5%)	59(36.2%)		
**Following a weight loss diet**:					
Yes	68(27.2%)	35(21%)	38(23.3%)		0.3
No	182(72.8%)	132(79%)	125(76.7%)	2.2	
** A number of meals and snacks per day**:					
Increase	106(42.4%)	58(34.7%)	63(38.7%)		
Decrease	51(20.4%)	32(19.2%)	25(15.3%)	5.6	0.2
No change	93(37.2%)	77(46.1%)	75(46%)		
**Fast food intake**:					
Increase	92(36.8%)	39(23.4%)	45(27.6%)		
Decrease	81(32.4%)	63(37.7%)	58(35.6%)	9.5	0.05
No change	77(30.8%)	65(38.9%)	60(36.8%)		
**The frequency of eating or the urge to eat sweets**:					
Increase	115(46%)	71(42.5%)	64(39.3%)		
Decrease	58(23.2%)	30(18%)	39(23.9%)		
No change	77(30.8%)	66(39.5%)	60(36.8%)	5.1	0.3
**Urge to drink coffee and tea**:					
Increase	134(53.6%)	78(46.7%)	87(53.4%)		
Decrease	40(16%)	20(12%)	25(15.3%)	6.2	0.2
No change	76(30.4%)	69(41.3%)	51(31.3%)		
**Amount of water intake**:					
Increase	136(54.4%)	83(49.7%)	91(55.8%		
Decrease	48(19.2%)	19(11.4%)	23(14.1%)	10.1	0.04*
No change	66(26.4%)	65(38.9%)	49(30.1%)		

**Table 3:** Comparison between employment and emotional eating scale and perceived stress scale among the studied participants

The scale	employment			X^2^	p
	Student	Employee	Staff		
**Emotional eating scale** A very healthy relationship with food	77(30.8%)	70(41.9%)	50(30.7%)		
Moderate emotional eating	127(50.8%)	71(42.5%)	89(54.6%)	7.9	0.09
High emotional eating	46(18.4%)	26(15.6%)	24(14.7%)		
**Perceived stress scale**					
Low stress	16(6.4%)	21(12.6%)	28(17.2%)		
Moderate stress	210(84%)	135(80.8%)	124(76.1%)	12.9	0.01*
High stress	24(9.6%)	11(6.6%)	11(6.7%)		

**X**^**2**^ **= Chi-square test**

*p value < 0.05.

**Table 4:** Correlation between emotional eating scale and perceived stress scale, sex, BMI and weight

Perceived stress scale	Emotional eating score			p	r
	A very healthy relationship with food	Moderate emotional eating	High emotional eating		
**Low stress**	30(15.2%)	32(11.1%)	3(3.1%)		
**Moderate stress**	152(77.2%)	235(81.9%)	82(85.4%)	0.001*	0.13
**High stress**	15(7.6%)	20(7%)	11(11.5%)		
**Sex**:					
**Male**	109(55.3%)	171(59.6%)	62(64.6%)	0.1	0.06
**Female**	88(44.7%)	116(40.4%)	34(35.4%)		
**BMI**	26 ± 3.9	25.8 ± 3.7	26.8 ± 4.4	0.1	1.6
**Weight**	75.4 ± 13.7	76.8 ± 12.6	79.9 ± 13.7	0.007*	0.11

R = pearson correlation.

*p value < 0.05.

**Table 5:** Predictors of emotional eating score among participants

	Unadjusted			adjusted		
Predictors	B ± SE	Std. Beta	P value	B ± SE	Std. Beta	P value
**Age**	0.02 ± 0.05	0.03	0.7	0.1 ± 0.6	0.05	0.9 0.8
**Gender**	-0.05 ± 0.8	-0.04	0.5	-0.07 ± 0.9	-0.03	
**BMI**	0.04 ± 0.07	0.2	0.5	0.2 ± 0.1	0.1	0.3 1.1 0.3 0.5 0.1
**Education**	-0.009 ± 0.1	-0.007	0.9	-0.1 ± 0.2	0.5	
**Employment**	0.08 ± 0.07	0.1	0.2	0.1 ± 0.08	0.4	
**Residence**	0.04 ± 0.06	0.03	0.5	0.7 ± 0.07	0.02	
**Marital status**	0.3 ± 0.1	0.2	0.02*	0.6 ± 0.2	0.05	
**Family income** **COVID-19 infection** **PSS score**	-0.01 ± 0.05 -0.6 ± 1.4 0.2 ± 0.1	-0.01 -0.02 0.11	0.8 0.6 0.006*	-0.3 ± 0.7 -0.4 ± 1.4 0.3 ± 0.1	-0.4 -0.01 0.1	0.7 0.3 0.001*

## Discussion

It has been known that the lockdown may result in unavoidable boredom and stress, which can lead to disrupted eating patterns and frequent snacking on high-calorie foods [27]. The combination of disturbed eating patterns and long hours of confinement, which is one of the effects of social isolation, is a major risk factor for all-cause and cardiovascular mortality, as well as an increased risk of obesity, diabetes, hypertension, and cardiovascular diseases [28].

In our study, More than two-fifths of students, employees, and staff (45.2%, 45.5%, and 44.2%, respectively) stated that their weight increased as a result of the lockdown. 72.8 of students, 79% of employees, and 76.9%of staff did not follow a weight loss program. Regarding the weight changes that occurred during the period of the COVID-19 lockdown. More than one-third of participants (42.4% of students, 34.7% of employees, and 38.7% of staff) consumed more meals and snacks daily. Additionally, fast food consumption increased for 36.8% of students, 23.4% of employees, and 27.6% of staff. These data indicate a significant presence of unhealthy eating behaviors during the COVID-19 pandemic.

Similarly, Ramalho et al., in their study among Portuguese adults found that during the period of COVID-19 lockdown; 38.2% of their participants showed an increase in their weight and only 15.7% of them reported a decrease in their weight during the same period [29]. Moreover, other studies from the United States, France, Italy, and Kuwait showed weight gain among their participants during the COVID-19 lockdown [30–33].

On the contrary, 40.1% of adults in the United Arab Emirates maintained their weight during the pandemic lockdown and 20.9% of them reported weight loss [34]. These differences among people in different countries regarding their weight changes may be attributed to the differences in the eating habits of people and the differences in food components and cooking methods in different countries all over the world.

As regards, perceived stress, more than three-quarters of students, employees, and staff reported moderate levels of stress (84%, 80.8%, and 76.1% respectively). This is to the results of Ramalho et al., as they found that most of their participants experienced mild to severe stress (87%) [29]. Also, Güneşer et al., found that a significant proportion of their participants found to have a moderate level of stress during the period of lockdown [7]. Additionally, Wang et al., demonstrated that more than half of their Chinese participants experienced moderate to severe psychological impact during the COVID-19 lockdown [35]. Moreover, Al-Musharaf, S., 2020, found in her study among young Saudi women that 71% were moderately stressed and 12.5% were severely stressed [36]. It is similar to data from surveys conducted in Saudi Arabia, Spain, India, and China during the COVID-19 pandemic [37–40].

In the present study, 50.8% of students, 42.5% of employees, and 54.6% of staff were in the moderate emotional eating groups. Di Renzo et al., in their Italian online survey, found that nearly half of their respondents reported that they felt anxious about their eating habits during the COVID-19 quarantine as 48.7% of them declared that they use food in response to anxious feelings and 55.1% of them need to increase their food consumption to feel better [32]. Also, in a study done in Australia to assess the impact of COVID-19 lockdown on changing the eating behaviors of the Australian population, Phillipou et al., found that there was a 34.6% in binge eating behaviors among the adult population when compared with a pre-pandemic period [41].

In the current study, we found that there was a positive correlation between total emotional eating score, perceived stress scale score, and weight. When the multiple linear regression analysis was done, we found that total emotional eating scores were positively correlated with the perceived stress scale score. Similarly, Ramalho et al., in their study found among Portuguese individuals, found that the higher psychosocial impact of lockdown during the COVID-19 crisis was positively associated with anxiety, stress levels, and emotional eating with a positive correlation between stress and the psychosocial impact caused by lockdown during COVID-19 and the prevalence of emotional eating among their participants [29]. Among Italian adults, Guerrini Usubini et al., found in their study that the total effect of psychological stress during the COVID-19 lockdown was significant on emotional eating [42]. Also, Cecchetto et al., found that emotional eating among the Italian population was significantly increased with higher levels of stress, anxiety, and depression during the COVID-19 lockdown [43]. In constant with this, Güneşer and Him, in their study on college students, found that there was a low positive correlation between perceived stress score and emotional eating score but with no statistical significance [7]. All of these findings were in accordance with previous studies that reported that emotional eating was triggered by stress, depression, anxiety, and emotional dysregulation [44].

In assessing if the demographic variables were predictors of emotional eating, we found in the current study that weight was positively correlated with the emotional eating score. We found no significant association between emotional eating and sex as males reported more emotional eating than females while females were found to have a healthy relationship with food compared to males. This may be because females have more concern about their weight and worried about weight gain than males so they do their best to be not involved in emotional eating.

In contrary with our findings, Guerrini Usubini et al., and Di Renzo et al., found that only sex had a significant association with emotional eating in their studies on the Italian population. Both studies found that females were more prone to emotional eating than males as they need to increase their intake of food to feel better and also they used food to relieve their anxious state [32, 42]. On comparing the perceived stress score and an emotional eating score of participants about their gender Güneşer and Him revealed in their study that both scores of females were significantly higher than males [7].

In another study, Kinasz et al. found that females had higher scores in terms of emotional eating while males had higher scores regarding conscious eating [45]. A significant difference was also found by Guerrini Usubini et al., between Italian males and females regarding their scores of emotional eating as they found that females reported higher levels of emotional eating than males [42]. This may be explained by the fact that females are more emotional than males and the pattern of their food intake can be affected easily by their emotional state more than males as they may increase their food intake as a way to relieve their stress. Unfortunately, these relationships are not consistently documented in the literature, highlighting the need for more studies to understand the role of gender in the COVID-19 psychological impact and its links with eating disorders [46].

On assessing the other sociodemographic and clinical characteristics as predictors of emotional eating, we didn’t find any significant relationship between the age or body mass index of our participants and their emotional eating scores. It may be due to the nature of our sample, which comprised young people of nearly normal BMI. Similar results were observed by Guerrini Usubini et al., 2021 among the Italian population, and his findings were explained by the theory of inconsistent associations, which had already been established [42].

Different findings were found by Bemanian, et al., in their study as they reported that emotional eating among their participants was affected by their age as its prevalence was least among the oldest age groups [47]. Moreover, Coulthard et al., found in their study that higher scores of emotional eating during the COVID-19 lockdown were associated with BMI as it was more prevalent among those with higher BMI [48]. Furthermore, Di Renzo et al., found a significant association between emotional overeating and the age, gender, and BMI of their participants during COVID-19 home confinement [32]. Also, Modrzejewska, et al., in their study among polish adults, and Bemanian et al., in their study on adults in Norway found that females and those with higher BMI were associated with higher levels of emotional overeating [47, 49].

We observed a positive correlation between weight and the total score of emotional eating. Several studies have found that obese people eat emotionally [50–52]. The researchers stated that intense emotions such as anxiety, restlessness, anger, fear, and excitement produce major changes in eating behaviors by increasing motivation to eat and the amount of food ingested [53–55]. Because we know that COVID-19 causes severe stress and anxiety in our lives and alters nearly every aspect of our lifestyle, it is obvious that it will influence eating habits, especially in people who already eat a lot. As a result, obese persons, particularly those who detect an increase in food consumption or emotional eating disorders during the COVID-19 quarantine, should seek professional help to avoid unnecessary weight gain [56]. Research at the Obesity Unit investigated weight changes and BMI before and after the COVID-19 lockdown. They discovered that following the COVID-19 lockdown, weight gain and BMI increased by approximately + 1.51 kg and + 0.58 kg/m2, respectively [57]. According to the researchers, 48.6% of the individuals gained weight. In 57.8% of the participants, the frequency and habits of meals altered, and the time spent in the kitchen increased [32].

## Conclusion

Our study found an increase in emotional overeating to cope with the COVID-19 pandemic and the negative feelings associated with it. The majority of participants were in the moderate emotional eating groups. More than three-quarters of students, employees, and staff reported a moderate level of stress. The total emotional eating score was positively correlated with the total perceived stress scores and weight. Follow-up surveys are needed to give further insight into emotional overeating, demographic characteristics, eating motivations, and COVID-19-related stress.

## Recommendations

Psychological counseling and intervention should be implemented as an effective option to deal with stressors that can be created by subsequent COVID-19 pandemic waves and further periods of lockdown. Behavioral therapy may be very effective for improving emotional balance and regulation and improving the coping strategies of people during periods of pandemics and lockdowns.

## Data Availability

The datasets generated and/or analyzed during the current study are not publicly available due to the belonging of data to the authors’ affiliated institute (Faculty of Medicine -Tanta University ), as well as to assure the privacy of respondents’ information. But data would be available from the corresponding author on reasonable request.
